# Supplementary material for: Development and validation of novel prognostic models for zinc finger proteins-related genes in soft tissue sarcoma
Source: Aging (Albany NY). 2023 Apr 26;15(8):3171–90. doi: 10.18632/aging.204682 (PMC10188339; doi:10.18632/aging.204682)
Supplement: Supplementary Figures [file aging-15-204682-s001.pdf]

## SUPPLEMENTARY FIGURES

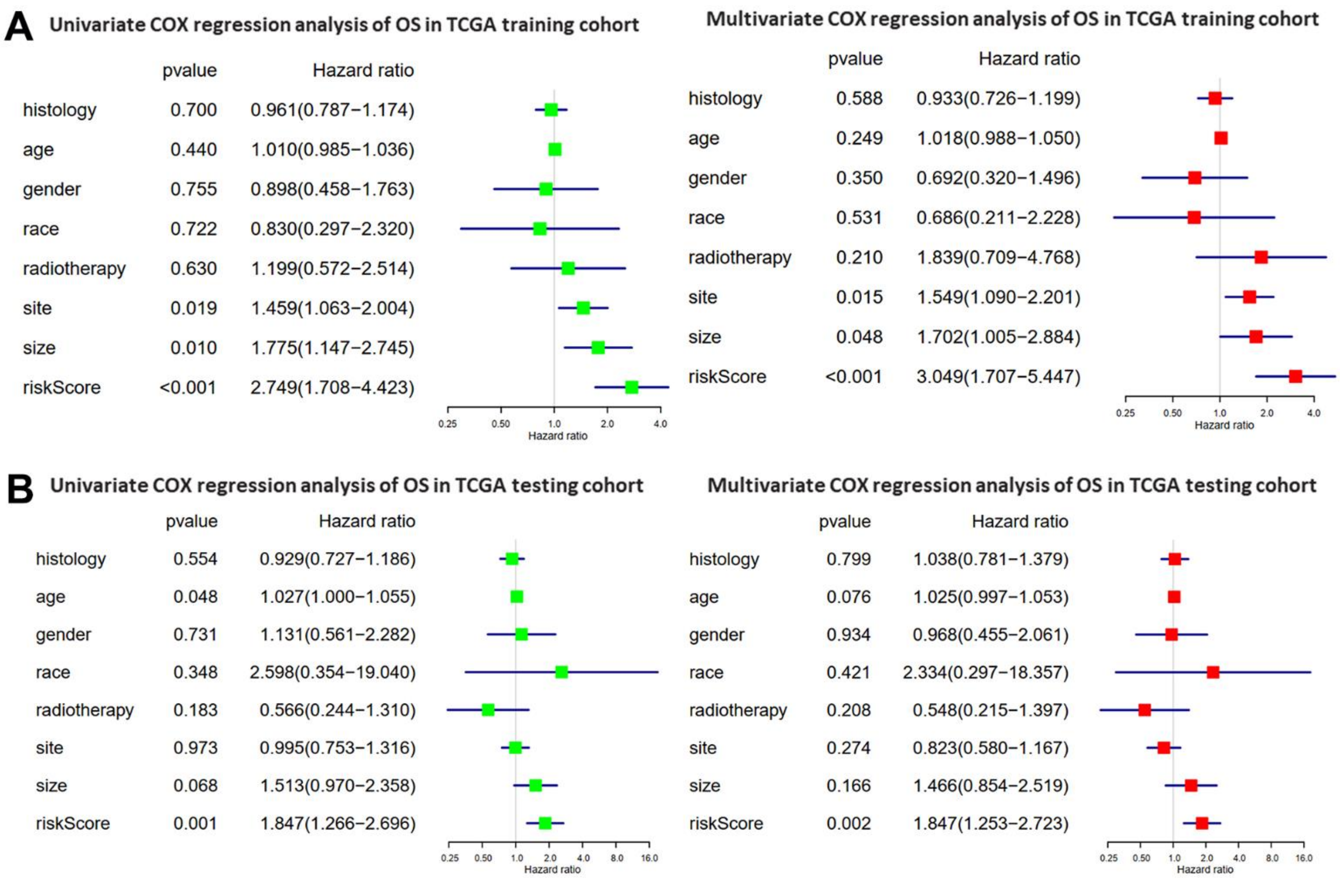


**Supplementary Figure 1.** Univariate and multivariate Cox regression analysis of OS in TCGA training (**A**) and testing cohorts (**B**).

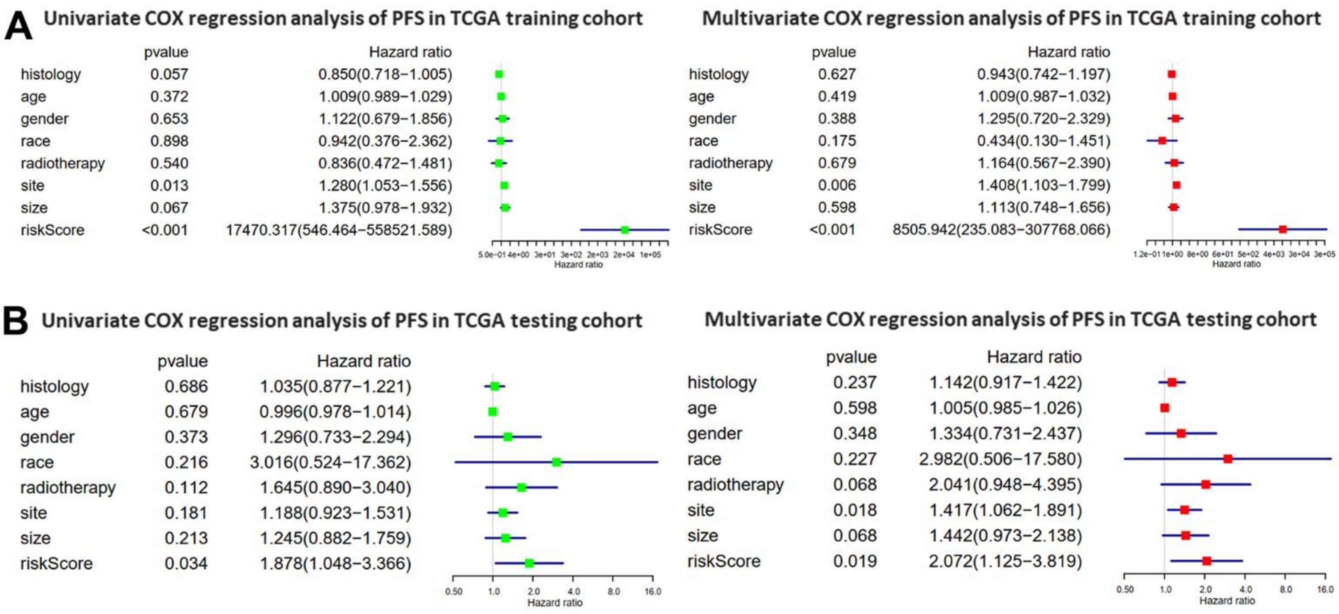


**Supplementary Figure 2.** Univariate and multivariate Cox regression analysis of PFS in TCGA training (**A**) and testing cohorts (**B**).

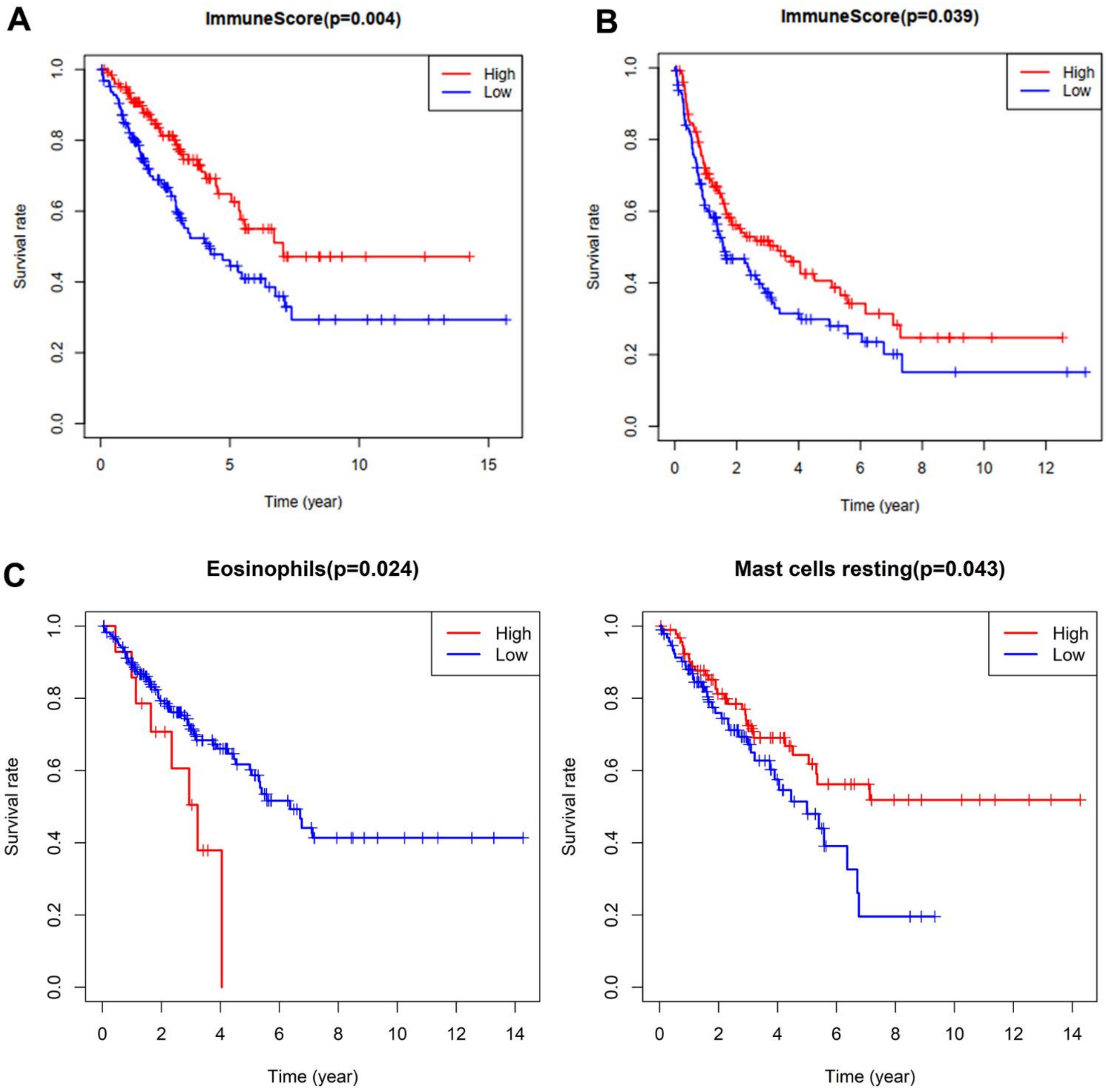


**Supplementary Figure 3. Survival curve of immune score and tumor-infiltrating immune cells.** OS (**A**) and PFS (**B**) curve of patients with high- or low- immune scores in the TCGA cohort. (**C**) OS curve of eosinophils and mast cells resting in the TCGA cohort.
