# Supplementary material for: Development and validation of novel prognostic models for zinc finger proteins-related genes in soft tissue sarcoma
Source: Aging (Albany NY). 2023 Apr 26;15(8):3171–90. doi: 10.18632/aging.204682 (PMC10188339; doi:10.18632/aging.204682)
Supplement: Supplementary Table 2 [file aging-15-204682-s003.pdf]

Supplementary Table 2. The sequences of primer and siRNA oligonucleotides.

**ZNF141**	**F**	**5'- GACGGTCCACAGATCGGAG-3'**
	R	5'- TGACTCAGGAGCGAAAATTGTT -3'
GAPDH	F	5'- ACAACTTTGGTATCGTGGAAG -3'
	R	5'- GCCATCACGCCACAGTTTC -3'
si-1	SS	5'-GUGUCAAAGUUGUUAGUAA-3'
	AS	5'-UUACUAACAACUUUGACAC-3'
si-2	SS	5'-GAGAUGUGAUGUUGGAGAA-3'
	AS	5'-UUCUCCAACAUCACAUCUC-3'
